# Supplementary material for: Isotope engineering for spin defects in van der Waals materials
Source: Nat Commun. 2024 Jan 2;15:104. doi: 10.1038/s41467-023-44494-3 (PMC10761865; doi:10.1038/s41467-023-44494-3)
Supplement: Supplementary file 1 — Supplementary Information [file 41467_2023_44494_MOESM1_ESM.pdf]

Supplementary Information and Extended Figures: Isotope Engineering for Spin Defects in van der Waals Materials

Ruotian Gong,[1,*] Xinyi Du,[1,*] Eli Janzen, [2] Vincent Liu,[3] Zhongyuan Liu,[1] Guanghui He,[1] Bingtian Ye,[3] Tongcang Li,[4,5] Norman Y. Yao,[3] James H. Edgar,[2] Erik A. Henriksen,[1,6] Chong Zu[1,6,†]

[1]Department of Physics, Washington University, St. Louis, MO 63130, USA
[2]Tim Taylor Department of Chemical Engineering, Kansas State University, Manhattan, KS 66506, USA
[3]Department of Physics, Harvard University, Cambridge, MA 02138, USA
[4]Department of Physics and Astronomy, Purdue University, West Lafayette, Indiana 47907, USA
[5]Elmore Family School of Electrical and Computer Engineering, Purdue University, West Lafayette, IN 47907, USA
[6]Institute of Materials Science and Engineering, Washington University, St. Louis, MO 63130, USA
[*]These authors contributed equally to this work
[†]To whom correspondence should be addressed; E-mail: zu@wustl.edu

(Dated: December 12, 2023)

CONTENTS

Supplementary Note 1. Experimental Setup 1

Supplementary Note 2. The Fourier Transformation Method for Calculating ESR Spectra 2

Supplementary Note 3. Differential Measurement Scheme 3

Supplementary Note 4. Magnetic Field Sensitivity Estimate 3

Supplementary Note 5. Interaction Hamiltonian for V_B^- and nearby Nuclear Spins 4

Supplementary Note 6. Enhanced Gyromagnetic Ratio for nuclear spin driving 5
- Supplementary Note 6.1. Derivation of effective nuclear gyromagnetic ratio 5
- Supplementary Note 6.2. Experimental characterization of effective nuclear gyromagnetic ratio 6
- Supplementary Note 6.3. Simulation of Nuclear Spin Resonance and Rabi Oscillations 7

Supplementary Note 7. Optical Characterizations of V_B^- in hBN 8

Supplementary Note 8. Extract sample thickness from the microscope optical image via color cycle fitting 8

References 15

Supplementary Note 1. EXPERIMENTAL SETUP

We characterize the spin properties of V_B^- ensembles in both $h^{10}B^{15}N$ and hBN_{nat} using a homebuilt confocal laser microscope. A 532 nm laser (Millennia eV High Power CW DPSS Laser) is used for both V_B^- spin initialization and detection. The laser is shuttered by an acousto-optic modulator (AOM, G&H AOMO 3110-120) in a double-pass configuration to achieve $> 10^5 : 1$ on/off ratio. An objective lens (Mitutoyo Plan Apo 100x 378-806-3) focuses the laser beam to a diffraction-limited spot with diameter ~ 0.6 μm and collects the V_B^- fluorescence. The fluorescence is then separated from the laser beam by a dichroic mirror and filtered through a long-pass filter before being detected by a single photon counting module (Excelitas SPCM-AQRH-63-FC). The signal is then processed by a data acquisition device (National Instruments USB-6343). The objective lens is mounted on a piezo objective scanner (Physik Instrumente PD72Z1x PIFOC), which controls the position of the objective and scans the laser beam vertically. The lateral scanning is performed by an X-Y galvanometer (Thorlabs GVS212).

To isolate an effective two-level system $|m_s = 0, -1\rangle$ or $|m_s = 0, +1\rangle$, we position a cylindrical N40 Neodymium permanent magnet with diameter 0.750" (19.05mm) and length 1" (25.40mm) directly on top of the sample to create an strong external magnetic field along the c-axis of the hBN lattice. Under this magnetic field, the $|m_s = \pm 1\rangle$ sublevels

of the V_B^- are separated due to the Zeeman effect and exhibit a splitting $2\gamma_e B$, where $\gamma_e = (2\pi) \times 2.8$ MHz/G is the gyromagnetic ratio of the V_B^- electronic spin. A resonant microwave drive is applied to address the transition between the two electronic spin sublevels, whose frequency depends on the strength of the external magnetic field and is probed via the electron spin resonance (ESR) measurement. Using a translation stage, we are also able to adjust the alignment angle of the magnetic field to explore its effect on nuclear spin polarization. We first move the $\hat{z}$ axis of the translation stage to approach the required external magnetic field strength $B \sim 760$ G for esLAC and then carefully adjust the $\hat{x}$ and $\hat{y}$ axes of the translation stage so that the nuclear polarization is maximized. We investigate the relationship between the alignment of B and the nuclear polarization by walking the $\hat{x}$ axis of the translation stage incrementally and recording the corresponding ESR signal to characterize polarization. By simulating the magnetic field vector of the specific magnet we use, we are able to map the translation distance to the alignment angle assuming the starting maximum point corresponds to $0°$.

The microwave driving field is generated by mixing the output from a microwave source (Stanford Research SG386) and an arbitrary wave generator (AWG, Chase Scientific Wavepond DAx22000). The AWG we use has a sampling rate of 2 GHz (0.5 ns temporal resolution), sufficiently fast to generate high-fidelity pulses to control the spin state of V_B^- ensembles. Specifically, a high-frequency signal at $(2\pi) \times 1$ GHz to $(2\pi) \times 6$ GHz from the microwave source is combined with a $(2\pi) \times 0.125$ GHz signal from the AWG using a built-in in-phase/quadrature (IQ) modulator so that the sum frequency is resonant with one of the $|m_s = 0\rangle \Longleftrightarrow |m_s = \pm 1\rangle$ transitions. By modulating the amplitude, duration, and phase of the AWG output, we can control the strength, rotation angle, and axis of the microwave pulses. For radio-frequency driving fields, we use another signal generator (Stanford Research SG384). Both the RF and microwave signals are shuttered by a switch (Minicircuits ZASWA-2-50DRA+) to prevent any leakage. The microwave and RF signal are amplified by amplifiers (Mini-Circuits ZHL-15W-422-S+ for microwave and LZY-22+ for RF) and delivered to the hBN sample through a coplanar waveguide. For nuclear resonance measurements, we combine the two signals using a 2 way power splitter (Mini-Circuits ZAPD-30-S+). The equipment is gated through a programmable multi-channel pulse generator (SpinCore PulseBlasterESR-PRO 500) with a 2 ns temporal resolution.

To investigate the temperature dependence of the spin relaxation timescales, we load both $h^{10}B^{15}N$ and hBN_{nat} sample into an optical accessible low-vibration cryostat (Four Nine Design SideKick Cryogenic Systems). We use a PID cryogenic temperature controller (Lake Shore Model 336) that operates from 4 K up to 350 K for the temperature control.

Supplementary Note 2. THE FOURIER TRANSFORMATION METHOD FOR CALCULATING ESR SPECTRA

To further illustrate the methodology used in obtaining the simulated ESR spectra for hBN samples with different isotope choices (Main text Fig. 1c and Fig. 3), we detail the calculation of spectral density, $S(\omega)$, which describes the density of states in the energy spectrum. We define $S(\omega)$ as a sum of Dirac functions over all possible $m_{\mathcal{I}}$, each centered at the respective energy $\sum_j A_{zz}^j m_{\mathcal{I}}^j$, where $m_{\mathcal{I}}^j$ represents the nuclear spin magnetic quantum number of the jth nuclear spin $\mathcal{I}^j$. This can be represented as

$$S(\omega) = \sum_{\text{all possible } \{m_{\mathcal{I}}\}} \delta(\omega - \sum_j A_{zz}^j m_{\mathcal{I}}^j). \tag{1}$$

We then establish the Fourier transform and its inverse as

$$\tilde{f}(\omega) = \mathcal{F}[f(t)] = \int_{-\infty}^{+\infty} f(t) e^{i\omega t} \mathrm{d}t \tag{2}$$

$$f(t) = \mathcal{F}^{-1}[\tilde{f}(\omega)] = \frac{1}{2\pi} \int_{-\infty}^{+\infty} \tilde{f}(\omega) e^{-i\omega t} \mathrm{d}\omega. \tag{3}$$

We can rewrite $S(\omega)$ in terms of the Fourier transform as

$$S(\omega) = \sum_{\text{all possible } \{m_{\mathcal{I}}\}} \frac{1}{2\pi}\mathcal{F}\{e^{-i\sum_j A^j_{zz} m^j_{\mathcal{I}} t}\}$$
$$= \sum_{\text{all possible } \{m_{\mathcal{I}}\}} \frac{1}{2\pi}\mathcal{F}\{\prod_j e^{-iA^j_{zz} m^j_{\mathcal{I}} t}\} \tag{4}$$
$$= \frac{1}{2\pi}\mathcal{F}\{\prod_j \sum_{-\mathcal{I}^j \le m^j_{\mathcal{I}} \le \mathcal{I}^j} e^{-iA^j_{zz} m^j_{\mathcal{I}} t}\}.$$

By employing these techniques, we circumvent computationally expensive calculations involving massive summations, replacing them with a tolerable number of steps involving summations, sequential multiplications, and the Fast Fourier Transform (FFT).

Supplementary Note 3. DIFFERENTIAL MEASUREMENT SCHEME

To accurately probe the spin dynamics of V_B^- , we utilize a robust differential measurement scheme illustrated in Fig. 5 [1]. Specifically, after letting the spin system reach charge state equilibration for 20 μs without any laser illumination (I), we apply a 5 μs laser pulse (532 nm) to initialize the spin state of V_B^- (II), followed by the measurement pulse sequences (III). Taking spin echo coherent measurements on the $|0\rangle$ and $|-1\rangle$ state as an example, we first apply a $\frac{\pi}{2}$-pulse along the $\hat{y}$ axis to prepare the system in a superposition state $\otimes_i \frac{|0\rangle_i + |-1\rangle_i}{\sqrt{2}}$, and then let it evolve for time t. A refocusing π-pulse along the $\hat{x}$ axis at time $t/2$ is used to decouple the spin ensemble from static magnetic noise. A final $\frac{\pi}{2}$-pulse along the $-\hat{y}$ direction rotates the spin back to the $\hat{z}$ axis for fluorescence detection (IV) and the measured photon count is designated as the bright signal $S_B(t)$. By repeating the same sequence but with a final $\frac{\pi}{2}$-pulse along the positive $+\hat{y}$ axis before readout, we measure the fluorescence of an orthogonal spin state to be the dark signal $S_D(t)$. The difference between the two measurements $C(t) \equiv [S_B(t) - S_D(t)]/S_R(t)$ can faithfully represent the measured spin coherent dynamics of V_B^- , where $S_R(t)$ is a reference signal we measure at the end of the initialization laser pulse (II).

Supplementary Note 4. MAGNETIC FIELD SENSITIVITY ESTIMATE

The static magnetic field sensitivity of ESR measurements takes the form [2, 3]

$$\eta_{\mathrm{DC}} \approx \frac{2\pi}{\gamma_e\sqrt{R}}(\max|\frac{\partial C(\nu)}{\partial \nu}|)^{-1} \approx \frac{8\pi}{3\sqrt{3}}\frac{1}{\gamma_e}\frac{\Delta\nu}{C_m\sqrt{R}}, \tag{5}$$

where γ_e denotes the electron gyromagnetic ratio ($2\pi \times 2.8$ MHz/G), R the photon detection rate, $C(\nu)$ the ESR measurement contrast at microwave frequency ν, C_m the maximum contrast, and $\Delta\nu$ the FWHM linewidth assuming a single Lorenztian resonance. If we directly compare the fitted FWHM from V_B^- in $h^{10}B^{15}N$ and hBN_{nat} (main text Fig. 1c), we obtain a factor of ~ 1.8 improvement in sensitivity. However, directly comparing the maximum $\frac{\partial C(\nu)}{\partial \nu}$ of each spectrum more accurately reflects the sensitivity as the hyperfine levels largely overlap with each other in hBN_{nat} and one cannot resolve individual Lorenztian. In Fig 6a, we plot $\frac{\partial C(\nu)}{\partial \nu}$ against frequency for the corresponding ESR spectrum of V_B^- in $h^{10}B^{15}N$ and hBN_{nat} from main text Figure 1, where the curves are obtained by differentiating the fitted Lorentzians. Here, we find the steepest slopes are 8.2×10^{-11} Hz^{-1} and 3.0×10^{-10} Hz^{-1} for hBN_{nat} and $h^{10}B^{15}N$ respectively, and, with photon detection rate R being similar for both, the sensitivity enhancement now increases to $\sim$ 4-fold.

We remark that, when performing the ESR measurement in main text Figure 1c, we utilize small laser and microwave powers to avoid spectral broadening to resolve the intrinsic linewidth of the resonances. However, to optimize sensitivity, we may want to further fine-tune the laser and microwave powers. Specifically, increasing laser power leads to a higher count rate R but lower maximum contrast and broadened linewidth, while increasing microwave power can boost contrast but also cause power broadening of the resonances. By carefully adjusting the laser and microwave power while monitoring the ESR signal, we obtain the an improved spectrum for sensing (Fig 6b). In Figure 6c, we apply the same differentiation process to the new ESR spectrum, achieving a DC magnetic field sensitivity of

$\sim 10\ \mu$T Hz$^{-\frac{1}{2}}$ (with count rate $R \approx 2.7 \times 10^6$ photons per second).

For AC magnetic fields, the sensitivity is given by [3]

$$\eta_{\mathrm{AC}} \approx \frac{\pi}{2\gamma_e} \frac{1}{C_{\max} e^{-(\tau/T_2)} \sqrt{\mathcal{N}}} \frac{\sqrt{t_I + \tau + t_R}}{\tau}, \tag{6}$$

where $C_{\max}$ is the maximum T_2 measurement contrast, $\mathcal{N}$ is the average photon count collected in a single experimental sequence, τ is the full field interrogation time, and t_I and t_R are respectively the initialization and readout times. In our experiment, a minimum of $t_I + t_R \approx 2\ \mu$s is required to optically polarize and detect V_B^- , which is much larger than the measurement time $\tau = T_2$; in this limit, $\eta_{\mathrm{AC}} \propto 1/T_2$.

To perform T_2 measurements, we first record the Rabi oscillations for pulse control. Fig. 7a shows the Rabi signal of V_B^- in $h^{10}B^{15}N$ sample S1 when driving one of the two center resonances and in hBN_{nat} sample S4 when driving the center resonance. The spin echo measurement is described in the previous section Supplementary Note 3. To further improve the AC magnetic field sensitivity, we utilize a more advanced dynamical decoupling sequence, XY8, to extend the T_2 coherence time. Instead of a single refocusing π-pulse, XY8 employs a series of π-pulses with alternating phases to better decouple the V_B^- from the nearby nuclear spin bath (Fig. 7b). The measured XY8 coherence time $T_2^{\mathrm{XY}} \approx 501$ ns is more than two times the spin echo coherence time $T_2^{\mathrm{E}} \approx 200$ ns. Using the measured contrast, $C \approx 2\%$, and average count per sequence, $\mathcal{N} \approx 0.27$, where we estimate that the AC magnetic field sensitivity η_{AC} is optimized with a value $\approx 7\ \mu$T Hz$^{-\frac{1}{2}}$. The AC magnetic field sensing frequency is set by the corresponding filter function of the pulse sequence in the frequency domain [3]. For our measurement of XY8 with π-pulse length = 24 ns and pulse interval = 18 ns, the frequency of the detected AC signal is ~ 12 MHz.

Supplementary Note 5. INTERACTION HAMILTONIAN FOR V_B^- AND NEARBY NUCLEAR SPINS

In this section, we derive the Hamiltonian governing the interaction between the V_B^- electronic spin and nearby nuclear spins. The V_B^- electronic spin-1 operators can be written as

$$S_z = \begin{bmatrix} 1 & 0 & 0 \\ 0 & 0 & 0 \\ 0 & 0 & -1 \end{bmatrix}, \ S_x = \frac{1}{\sqrt{2}} \begin{bmatrix} 0 & 1 & 0 \\ 1 & 0 & 1 \\ 0 & 1 & 0 \end{bmatrix}, \ S_y = \frac{1}{\sqrt{2}i} \begin{bmatrix} 0 & 1 & 0 \\ -1 & 0 & 1 \\ 0 & -1 & 0 \end{bmatrix}. \tag{7}$$

We can also define the spin raising and lowering operators for the V_B^- electron spin as

$$S_+ = \sqrt{2} \begin{bmatrix} 0 & 1 & 0 \\ 0 & 0 & 1 \\ 0 & 0 & 0 \end{bmatrix} = S_x + iS_y, \ S_- = \sqrt{2} \begin{bmatrix} 0 & 0 & 0 \\ 1 & 0 & 0 \\ 0 & 1 & 0 \end{bmatrix} = S_x - iS_y, \tag{8}$$

and rewrite V_B^- spin operators in terms of the raising and lowering operators in the form

$$S_x = \frac{S_+ + S_-}{2}, \ S_y = \frac{S_+ - S_-}{2i}. \tag{9}$$

For nuclear spins, we consider spin-1/2 ^{15}N nuclei with spin operators

$$I_z = \frac{1}{2} \begin{bmatrix} 1 & 0 \\ 0 & -1 \end{bmatrix}, \ I_x = \frac{1}{2} \begin{bmatrix} 0 & 1 \\ 1 & 0 \end{bmatrix}, \ I_y = \frac{1}{2i} \begin{bmatrix} 0 & 1 \\ -1 & 0 \end{bmatrix}. \tag{10}$$

and spin ladder operators

$$I_+ = \begin{bmatrix} 0 & 1 \\ 0 & 0 \end{bmatrix} = I_x + iI_y, \ I_- = \begin{bmatrix} 0 & 0 \\ 1 & 0 \end{bmatrix} = I_x - iI_y, \tag{11}$$

Similarly, we can rewrite ^{15}N nuclear spin operators as

$$I_x = \frac{I_+ + I_-}{2}, \ I_y = \frac{I_+ - I_-}{2i}. \tag{12}$$

To expand the hyperfine interaction term between V_B^- and three ^{15}N nuclear spins — $\sum_{j=1}^{3} \mathbf{S}\mathbf{A}^j\mathbf{I}^j$ — we note the

hyperfine parameters tensor $\mathbf{A}$ for a single nuclear spin takes the form

$$\mathbf{A} = \begin{bmatrix} A_{xx} & A_{xy} & A_{xz} \\ A_{yx} & A_{yy} & A_{yz} \\ A_{zx} & A_{zy} & A_{zz} \end{bmatrix} = \begin{bmatrix} A_{xx} & A_{xy} & 0 \\ A_{yx} & A_{yy} & 0 \\ 0 & 0 & A_{zz} \end{bmatrix}. \tag{13}$$

Here the $\hat{z}$-axis is defined along the c-axis of hBN (perpendicular to the lattice plane, see main text Figure. 1), $\hat{x}$ and $\hat{y}$ lie in the lattice plane, with $\hat{x}$ oriented along one of the three in-plane nitrogen bonds. Due to the mirror symmetry of V_B^- with respect to the $\hat{x}-\hat{y}$ plane, the four terms A_{xz} A_{yz} A_{zx} A_{zy} vanish [4, 5]. We can then expand the hyperfine interacting Hamiltonian to its full form

$$\begin{aligned}
\sum_{j=1}^{3} \mathbf{S}\mathbf{A}^j\mathbf{I}^j &= \sum_{j=1}^{3}(A^j_{zz}S_zI^j_z + A^j_{xx}S_xI^j_x + A^j_{yy}S_yI^j_y + A^j_{xy}S_xI^j_y + A^j_{yx}S_yI^j_x) \\
&= \sum_{j=1}^{3}[A^j_{zz}S_zI^j_z + \frac{1}{4}A^j_{xx}(S_+ + S_-)(I^j_+ + I^j_-) - \frac{1}{4i}A^j_{yy}(S_+ - S_-)(I^j_+ - I^j_-) \\
&\quad + \frac{1}{4i}A^j_{xy}(S_+ + S_-)(I^j_+ - I^j_-) + \frac{1}{4i}A^j_{yx}(S_+ - S_-)(I^j_+ + I^j_-)] \\
&= \sum_{j=1}^{3}[A^j_{zz}S_zI^j_z + \frac{A_{xx} + A_{yy}}{4}(S_+I^j_- + S_-I^j_+) \\
&\quad + (\frac{A_{xx} - A_{yy}}{4} + \frac{A_{xy}}{2i})S_+I^j_+ + (\frac{A_{xx} - A_{yy}}{4} - \frac{A_{xy}}{2i})S_-I^j_-].
\end{aligned} \tag{14}$$

In the last step, we also use the fact that $A_{xy} = A_{yx}$ from the symmetry [4]. Finally, since $(S_+I^j_-)^\dagger = (S_-I^j_+)$ and $(S_-I^j_-)^\dagger = (S_-I^j_-)$, we can further simplify the expression to the form

$$\sum_{j=1}^{3} \mathbf{S}\mathbf{A}^j\mathbf{I}^j = \sum_{j=1}^{3}[A^j_{zz}S_zI^j_z + (A^j_1 S_+I^j_- + h.c.) + (A^j_2 S_+I^j_+ + h.c.)], \tag{15}$$

where we define $A^j_1 = \frac{1}{4}(A^j_{xx} + A^j_{yy})$ and $A^j_2 = \frac{1}{4}(A^j_{xx} - A^j_{yy}) + \frac{1}{2i}A^j_{xy}$.

Supplementary Note 6. ENHANCED GYROMAGNETIC RATIO FOR NUCLEAR SPIN DRIVING

Supplementary Note 6.1. Derivation of effective nuclear gyromagnetic ratio

In this section, we derive the enhanced effective gyromagnetic ratio of nuclear spins in the V_B^- center. For each nearest-neighbour ^{15}N nuclear spin, the hyperfine Hamiltonian is described by Eqn. 15, where by threefold rotational symmetry, $|A^j_1|$ and $|A^j_2|$ are the same for each nucleus. We note that this form does not hold in the excited state due to the breaking of threefold rotational symmetry by the Jahn-Teller effect.

To obtain the effective gyromagnetic ratio, it is instructive to first focus on a simplified case where the electronic spin couples to only one nuclear spin. The ground-state Hamiltonian can be written as a sum of secular and non-secular terms, denoted H_0 and δH respectively, with

$$H_0 = D_{gs}S_z^2 + \gamma_e B_z S_z - \gamma_n B_z I_z + A_{zz}S_zI_z, \tag{16a}$$

$$\begin{aligned}
\delta H &= \gamma_e B_{\mathrm{dr}}\left(\cos\theta S_x + \sin\theta S_y\right) - \gamma_n B_{\mathrm{dr}}\left(\cos\theta I_x + \sin\theta I_y\right) + (A_1S_+I_- + h.c.) + (A_2S_+I_+ + h.c.) \\
&= \frac{\gamma_e}{2}B_{\mathrm{dr}}\left(e^{i\theta}S_+ + e^{-i\theta}S_-\right) - \frac{\gamma_n}{2}B_{\mathrm{dr}}\left(e^{i\theta}I_+ + e^{-i\theta}I_-\right) + (A_1S_+I_- + h.c.) + (A_2S_+I_+ + h.c.)\,,
\end{aligned} \tag{16b}$$

where we have included microwave and RF drives with an transverse magnetic field B_{dr} at an angle θ to the $\hat{x}$ axis. Throughout our experiment, we always work at a magnetic field far away from ground-state anti-crossing (gsLAC, $B_z \approx 1240$ G), so that δH is suppressed due to the large splitting between the $|m_s = 0\rangle$ and $|m_s = \pm 1\rangle$ electronic spin levels of V_B^- . In this case, we can treat δH as a perturbation to H_0. Performing second-order perturbation theory,

we find that the coupling term between nuclear spin sublevels can be written as

$$-\frac{\gamma_n}{2}B_{\rm dr}\left(e^{i\theta}I_+ + e^{-i\theta}I_-\right) - \frac{\gamma_e}{D_{gs}\pm\gamma_e B_z}B_{\rm dr}\left[\left(A_1 e^{i\theta} + A_2^* e^{-i\theta}\right)I_- + h.c.\right] \tag{17}$$

for $|m_s = \pm 1\rangle$ states, and

$$-\frac{\gamma_n}{2}B_{\rm dr}\left(e^{i\theta}I_+ + e^{-i\theta}I_-\right) - \gamma_e\left(\frac{1}{D_{gs}+\gamma_e B_z} + \frac{1}{D_{gs}-\gamma_e B_z}\right)B_{\rm dr}\left[\left(A_1 e^{i\theta} + A_2^* e^{-i\theta}\right)I_- + h.c.\right] \tag{18}$$

for the $|m_s = 0\rangle$ state. Focusing on the $|m_s = -1\rangle$ level at esLAC where we perform the nuclear spin control and noting that $\gamma_n \ll \gamma_e \frac{|A_{1,2}|}{D_{gs}-\gamma_e B_z}$, we drop the first term and find that the effective nuclear spin Rabi frequency is $2\gamma_e B_{\rm dr}\frac{\left|A_1 e^{i\theta}+A_2^* e^{-i\theta}\right|}{D_{gs}-\gamma_e B_z}$, with a nuclear gyromagnetic ratio enhancement factor of $2\frac{\gamma_e}{\gamma_n}\frac{\left|A_1 e^{i\theta}+A_2^* e^{-i\theta}\right|}{D_{gs}-\gamma_e B_z}$.

In the V_B^- center, there are three nearest-neighbor nuclear spins instead of one. However, the perturbation theory analysis above is still valid for each individual nuclear spin because there are no terms that directly couple the nuclear spins of different nuclei. Thus, we can write the nuclear coupling terms in the $\left|m_s = -1, m_I^1, m_I^2, m_I^3\right\rangle$ basis as the matrix

$$\begin{bmatrix} 0 & \omega_3 & \omega_2 & 0 & \omega_1 & 0 & 0 & 0 \\ \omega_3^* & 0 & 0 & \omega_2 & 0 & \omega_1 & 0 & 0 \\ \omega_2^* & 0 & 0 & \omega_3 & 0 & 0 & \omega_1 & 0 \\ 0 & \omega_2^* & \omega_3^* & 0 & 0 & 0 & 0 & \omega_1 \\ \omega_1^* & 0 & 0 & 0 & 0 & \omega_3 & \omega_2 & 0 \\ 0 & \omega_1^* & 0 & 0 & \omega_3^* & 0 & 0 & \omega_2 \\ 0 & 0 & \omega_1^* & 0 & \omega_2^* & 0 & 0 & \omega_3 \\ 0 & 0 & 0 & \omega_1^* & 0 & \omega_2^* & \omega_3^* & 0 \end{bmatrix}, \tag{19}$$

where $\omega_j \equiv -\gamma_e B_{\rm dr}\frac{A_1^j e^{i\theta}+\left(A_2^j\right)^* e^{-i\theta}}{D_{gs}-\gamma_e B_z}$ corresponds to the j-th nuclear spin. The eigenvalues of this matrix are $\pm|\omega_1| \pm |\omega_2| \pm |\omega_3|$, leading to nuclear spin Rabi frequencies at $2\,||\omega_1| \pm |\omega_2| \pm |\omega_3||$. In general, $|\omega_i| \neq |\omega_j|$ for $i \neq j$ due to the different phases of the three A_2^j, so we predict that nuclear spin Rabi oscillations generically have four different frequencies of oscillation. This is qualitatively supported by the experimental data, which clearly shows oscillations of more than a single frequency.

We also note that the separate Rabi frequencies are highly dependent on the angle θ of the driving field, i.e. the relative angle between the RF magnetic field and the hBN crystal orientation. As an estimation, we take the averaged case and approximate $\left|A_1^j e^{i\theta_j} + \left(A_2^j\right)^* e^{-i\theta_j}\right| \sim \sqrt{\left|A_1^j\right|^2 + \left|A_2^j\right|^2} = \sqrt{|A_1|^2 + |A_2|^2}$ (as $\left|A_{1,2}^i\right| = \left|A_{1,2}^j\right|$ by symmetry). The nuclear spin Rabi frequencies then become $2\gamma_e B_{\rm dr}\frac{\sqrt{|A_1|^2+|A_2|^2}}{D_{gs}-\gamma_e B_z}$ (threefold degeneracy) and $6\gamma_e B_{\rm dr}\frac{\sqrt{|A_1|^2+|A_2|^2}}{D_{gs}-\gamma_e B_z}$, corresponding to a slow oscillation (in the general case, three slow oscillations) and a fast oscillation. We can approximately map the Rabi oscillation timescale to that of the slow oscillation, giving an effective nuclear gyromagnetic ratio of

$$\gamma_n^{\rm eff} = \frac{\Omega_n}{B_{\rm dr}} \approx 2\gamma_e\frac{\sqrt{|A_1|^2 + |A_2|^2}}{D_{gs} - \gamma_e B_z} = \frac{\gamma_e\sqrt{A_{xx}^2 + A_{yy}^2 + 2A_{xy}^2}}{\sqrt{2}(D_{\rm gs} - \gamma_e B_z)} \tag{20}$$

Supplementary Note 6.2. Experimental characterization of effective nuclear gyromagnetic ratio

To experimentally probe the effective gyromagnetic ratio of three nearest-neighbor ^{15}N nuclear spins, we first characterize the strength of the microwave pulse for electronic spin control and the RF pulse for nuclear spin control. In particular, we directly measure the amplitude of the microwave and RF pulses by connecting the output from the coplanar waveguide to an oscilloscope with a sampling rate at 100 GHz. In Figure. 8, we plot the measured waveform of the nuclear Rabi sequence, where we first apply a microwave π-pulse for electronic spin, a RF-pulse for nuclear spin, and then a final microwave π-pulse sequentially. By measuring the root-mean-square (RMS) voltage of each

pulse, we obtain the amplitude ratio between RF and microwave fields

$$R_{\text{volt}} = \frac{V_{\text{RF}}}{V_{\text{MW}}} \approx 2.6. \tag{21}$$

At these powers, the electronic and nuclear spin Rabi frequencies are measured to be $\Omega_e \approx (2\pi) \times 41.67$ MHz and $\Omega_n \approx (2\pi) \times 1.67$ MHz respectively. Since the Rabi frequency should be proportional to the driving field amplitude, after accounting their amplitude difference, the effective Rabi frequency for nuclear spin at the same power as electronic spin is $\Omega_n^{\text{eff}} = \Omega_n / R_{\text{volt}} \approx (2\pi) \times 0.64$ MHz. This leads to an effective nuclear spin gyromagnetic ratio

$$\gamma_n^{\text{eff}} = \frac{\Omega_n^{\text{eff}}}{\Omega_e} \times \gamma_e \approx (2\pi) \times 0.043 \text{ MHz/G} \tag{22}$$

Using our approximated nuclear gyromagnetic ratio from Eq. 20, we find that

$$\frac{\gamma_n^{\text{eff}}}{\gamma_e} \approx 99 \approx 2\frac{\gamma_e}{\gamma_n}\frac{\sqrt{|A_1|^2 + |A_2|^2}}{D_{gs} - \gamma_e B_z} = \frac{\gamma_e \sqrt{A_{xx}^2 + A_{yy}^2 + 2A_{xy}^2}}{\sqrt{2}(D_{\text{gs}} - \gamma_e B_z)}. \tag{23}$$

Using $\gamma_e/\gamma_n \approx 6487$ for ^{15}N nuclear spins and $D_{\text{gs}} - \gamma_e B_z \approx (2\pi) \times 1.39$ GHz at esLAC, we estimate the transverse hyperfine interaction term to be $\sqrt{A_{xx}^2 + A_{yy}^2 + 2A_{xy}^2} \approx (2\pi) \times 30$ MHz. We note that this value turns out to be much smaller than the value predicted by previous ab-initio calculations, $\sqrt{A_{xx}^2 + A_{yy}^2 + 2A_{xy}^2} \approx (2\pi) \times 142$ MHz [4, 5], by a factor of approximately 4.8. In the next section, we present more experimental evidence on the nuclear spin resonance and Rabi oscillation measurement to support our observation.

Supplementary Note 6.3. Simulation of Nuclear Spin Resonance and Rabi Oscillations

In this subsection, we provide further experimental and theoretical evidence on the discrepancy between the measured transverse hyperfine interaction from our experiment and previous ab-initio calculations. We begin by examining the nuclear spin resonance spectrum (main text Fig. 4b), as a large transverse hyperfine interaction will lead to visible shifts of each nuclear spin transition. To simulate the experimental process, we first generate the Hamiltonian including the V_B^- electronic spin ground state and the three nearest-neighbour ^{15}N nuclear spins (main text Eqn. 1 and Eqn. 14). Here, we have included all hyperfine terms in the Hamiltonian. After diagonalizing the Hamiltonian, we obtain the eigenenergies E_n and the associated eigenstates v_n of the system. The microwave and RF transitions from an initial state v_i to a final state v_f then has an energy $\Delta E = |E_f - E_i|$ and amplitude $T_{i\to f} = |\langle v_f|S_x|v_i\rangle| + |\langle v_f|S_y|v_i\rangle|$. We select the initial states v_i within the manifolds $m_s = -1$ and $\sum m_I = 1/2$ as in the experiment and calculate the corresponding transition energies and amplitudes to all possible final states. We focus on the frequency range of the nuclear spin transition ($\sim |A_{zz}| = (2\pi) \times 65.9$ MHz) and add a Lorentzian broadening with 2 MHz FWHM to each transition to better reproduce the experimental results.

If one directly uses the transverse hyperfine terms predicted by the ab-initio calculations, as shown in Figure 9, while the simulated nuclear spin spectrum still agrees with the experimental results at $B_z = 210$ G (Fig. 9b inset), it exhibits a significant shift at $B_z = 760$ G and cannot reproduce experimental results (Fig. 9b). In comparison, we simply rescale the transverse hyperfine terms A_{xx}, A_{yy}, A_{xy} values from ab-initio by a factor of 4.8 obtained by comparing theoretical and experimental Rabi oscillation timescales in the previous subsection. With these adjusted values, the simulated nuclear spin resonance spectra agree remarkably well with the experiments at at both $B_z = 210$ G and $B_z = 760$ G (main text Fig. 4c). We note that, at small magnetic fields, the effect of transverse hyperfine coefficients is highly suppressed by the large splitting between V_B^- $|0\rangle$ and $|-1\rangle$ spin states. As a result, the simulation with the calculated and adjusted values fit the experimental data eqully well. At large external magnetic field, however, the electronic spin splitting between $|0\rangle$ and $|-1\rangle$ become much smaller, and the effect of transverse hyperfine coefficients becomes evident.

We also perform simulations of the nuclear Rabi oscillation dynamics. Once again, simulations using transverse hyperfine values predicted by ab-initio yield significant disagreements from experiment, exhibiting Rabi oscillation signals at much faster oscillating frequencies than experimentally observed and indicating an exaggerated effective nuclear gyromagnetic ratio (Fig. 10a). On the other hand, simply rescaling the ab-initio values by a factor of 4.8 leads the simulated dynamics to display an oscillation with frequencies and patterns similar to the experiment, as shown in Figure 10b. We do not see total agreement due to the existence of undetermined parameters such as the angle θ as well as the specific values of A_1^j and A_2^j, which cannot be assumed to be simply rescaled from ab-initio calculations.

Still, the theoretical and experimental dynamics show remarkable qualitative agreement.

Supplementary Note 7. OPTICAL CHARACTERIZATIONS OF V_B^- IN HBN

To further characterize V_B^- in $h^{10}B^{15}N$, we first perform an optical saturation measurement by monitoring the fluorescence while increasing laser power. As we can see from Figure 11a, the fluorescence increases with laser power and gradully deviates from a linear relation around ~ 10 mW to saturation. Note that this saturation point is also similar to that of the nuclear spin polarization (Fig. 3c from the main text). This is not surprising since the nuclear spin polarize at esLAC through electronic spin and thus is also limited by the V_B^- polarization level. Additionally, we measure the photoluminescence spectrum of V_B^- in hBN_{nat}, $h^{10}B^{15}N$, and $h^{11}B^{15}N$ and found no significant difference (Fig. 11b).

Supplementary Note 8. EXTRACT SAMPLE THICKNESS FROM THE MICROSCOPE OPTICAL IMAGE VIA COLOR CYCLE FITTING

In this section, we provide the method we use for estimating the sample thickness from the microscope optical image analysis, which is a common empirical technique in 2D materials to estimate the flake thickness [6]. We first performed the optical imaging using a Zeiss AxioSkop optical microscope, with a fixed exposure time of 6 ms and automatic white balance adjustment. The hBN flakes were exfoliated onto Silicon wafers with 300 nm of thermal oxide, serving as a robust substrate. To extract the wavelength of individual hBN flakes, λ, we first convert the picture RGB values to the HSV (Hue, Saturation, Value) color space and estimate λ assuming a linear relationship between λ and Hue value.

In particular, we conducted AFM on ~ 30 separate hBN flakes with varying thicknesses, establishing a reliable mapping from the color of the hBN flakes under our microscope to the precise thickness (Fig. S12 in Supplemental Information). From the figure, we calibrate the thickness of the six flakes studied in our manuscript and summarize them in Supplementary Table 1.

	S1	S2	S3	S4	S5	S6
Thickness (nm)	64 ± 8	63 ± 8	63 ± 8	72 ± 8	70 ± 8	21 ± 8

Supplementary Table 1. Summary of the estimated flake thickness for the six hBN samples S1-6.

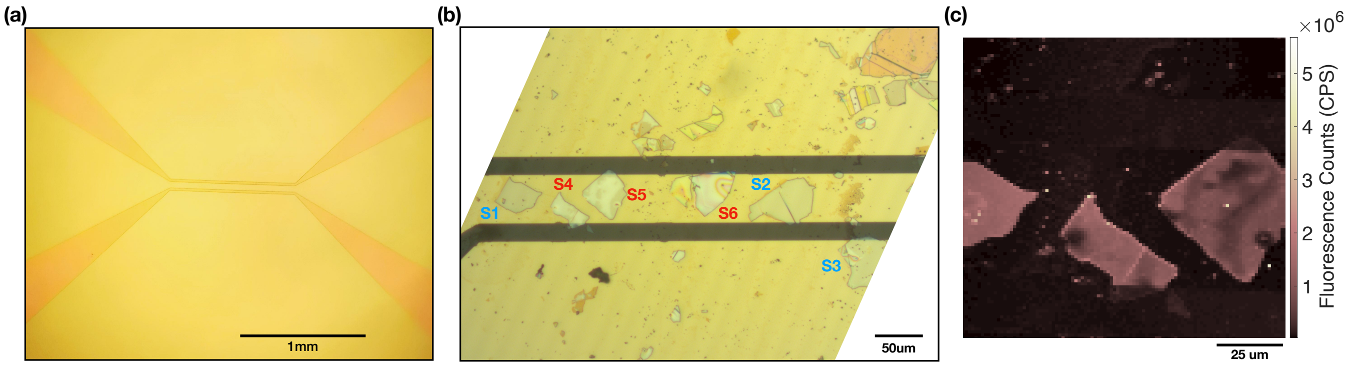

Supplementary Fig. 1. **Optical image of the coplanar waveguide** (a) Overview of the coplanar waveguide before transferring the hBN flakes. (b) Zoomed-in microscope image of the coplanar waveguide after transferring the hBN samples. The six flakes studied in this work are labeled as S1-S6. (c) Fluorescence confocal scan of sample S1, S4, and S5.

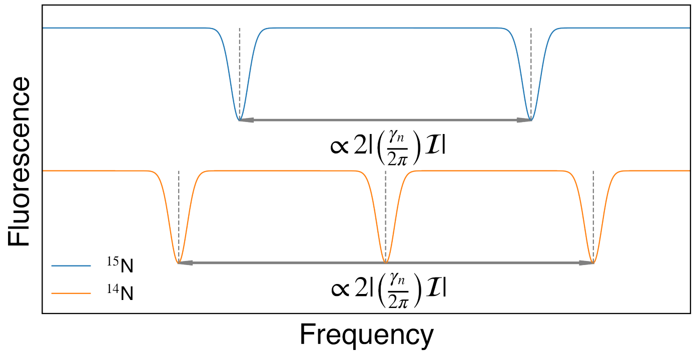

Supplementary Fig. 2. **Schematic ESR Spectrum** A schematic ESR spectrum of V_B^- coupling to a single ^{14}N (orange) or ^{15}N (blue) nuclear spin with a overall bandwidth of the transitions proportional to $2|(\frac{\gamma_n}{2\pi})\mathcal{I}|$.

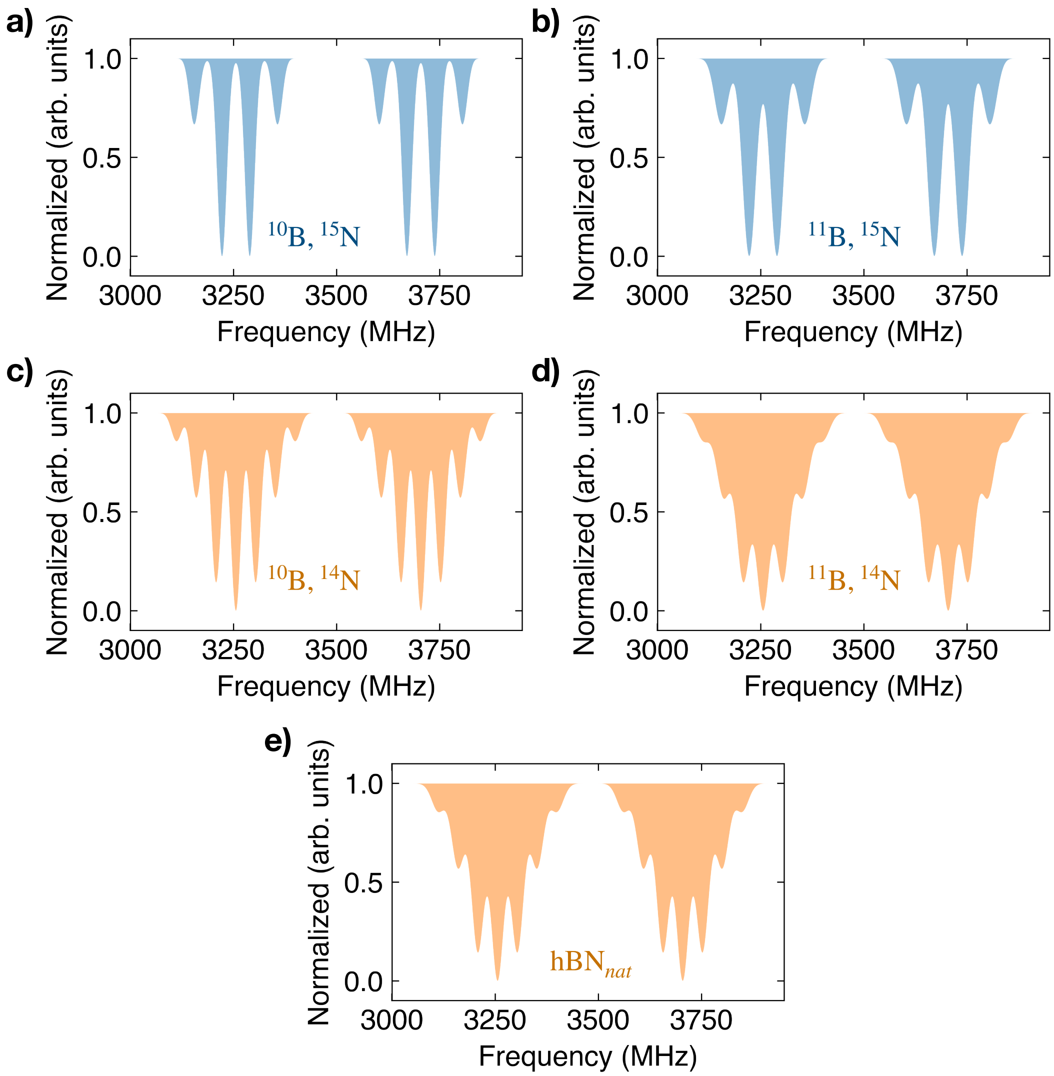

Supplementary Fig. 3. **Simulated ESR Spectra for Different Isotopes** Simulated ESR spectra of V_B^- in the four different possible isotopically purified hBN crystals (a) $h^{10}B^{15}N$, (b) $h^{11}B^{15}N$, (c) $h^{10}B^{14}N$, (d) $h^{11}B^{14}N$, and (e) in hBN_{nat}.

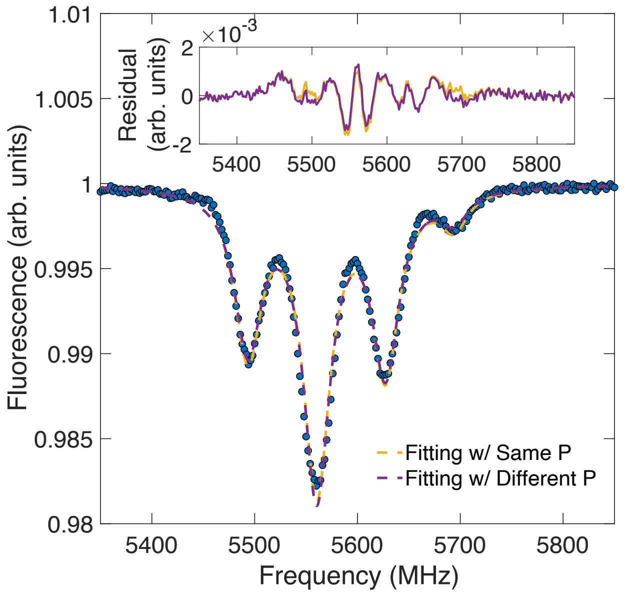

Supplementary Fig. 4. **Extracting Nuclear Spin Polarization** Fits of the ESR spectrum at esLAC using two different methods: assuming independently polarization probability P for all three ^{15}N nuclear spins, yielding the amplitude ratio $\{(1-P)^3 : 3P(1-P)^2 : 3P^2(1-P) : P^3\}$ (yellow dashed lines) and assuming two different nuclear polarization probabilities, P_1 and P_2, yielding amplitude ratio $\{(1-P_1)^2(1-P_2) : (1-P_1)^2P_2 + 2P_1(1-P_1)(1-P_2) : P_1^2(1-P_2) + 2P_1P_2(1-P_1) : P_1^2P_2\}$ (purple dashed lines). Inset: computed residuals between experimental data and two fits. Source data are provided as a Source Data file.

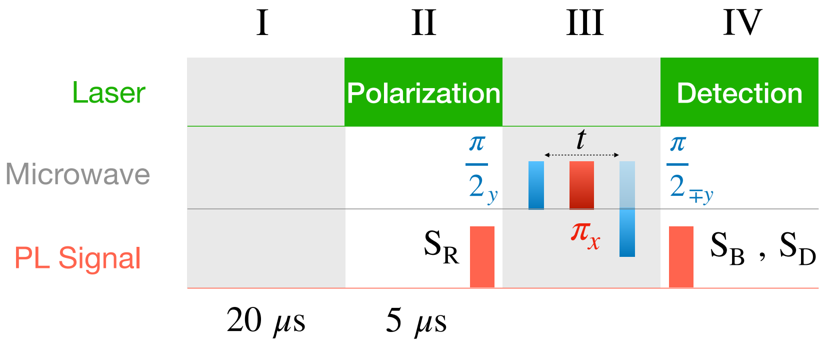

Supplementary Fig. 5. **Differential Measurement** Differential measurement sequence for spin echo. I: 20 μs wait time to reach charge state equilibration. II: 5 μs laser pulse to initialize the V_B^- spin to $|m_s = 0\rangle$, with the reference signal, $S_R(t)$, collected at the end of the laser pulse. III: microwave wave pulses for spin echo measurement; for the bright signal, a final $\frac{\pi}{2}$ pulse along the $-\hat{y}$ axis is applied; while for the dark signal, a final $\frac{\pi}{2}$ pulse along the $+\hat{y}$ axis is applied to rotate the spin to an orthogonal state. IV: laser pulse to detect the spin state.

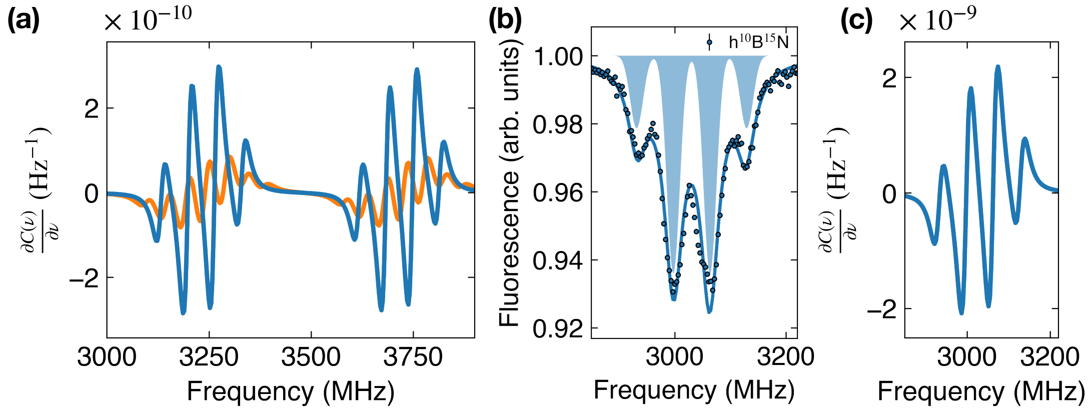

Supplementary Fig. 6. **Optimization of magnetic field sensitivity for ESR measurements** (a) Derivatives of the ESR spectra of V_B^- in $h^{10}B^{15}N$ and hBN_{nat} from main text Figure 1c, showing slopes with maximum at 8.2×10^{-11} Hz^{-1} and 3.0×10^{-10} Hz^{-1} respectively. (b) ESR spectrum of V_B^- in $h^{10}B^{15}N$ after optimizing the laser and microwave powers for better sensitivity. Error bars represent 1 s.d. accounting statistical uncertainties. (c) Derivatives of the fitted ESR spectrum in (b). Source data are provided as a Source Data file.

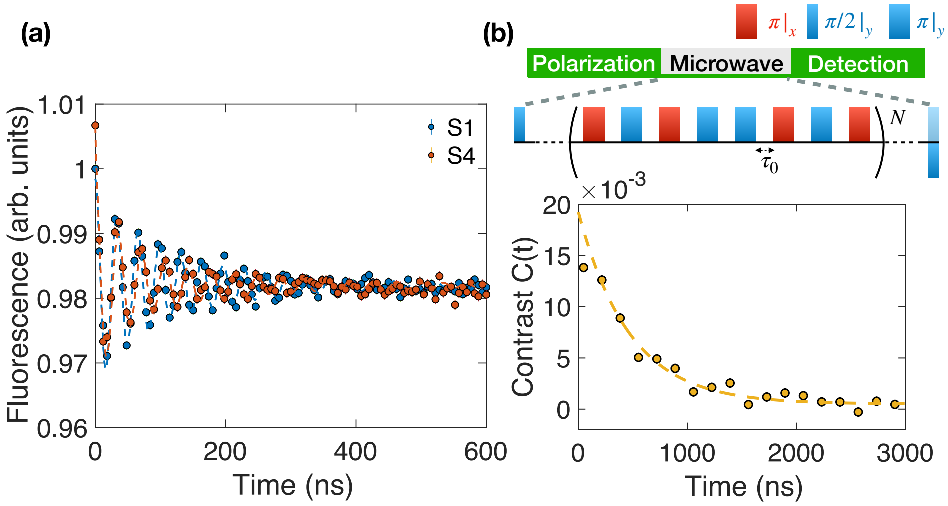

Supplementary Fig. 7. **Rabi Oscillations and XY8 T_2 Measurement** (a) Rabi oscillations recorded on $h^{10}B^{15}N$ sample S1 and hBN_{nat} sample S4 under the same microwave power. Error bars represent 1 s.d. accounting statistical uncertainties. (b) Coherent measurement of V_B^- in $h^{10}B^{15}N$ sample S3 using the XY8 pulse sequence. The sequence repeats itself every 8 pulses, and we take a measurement every 4 pulses to increase the number of data points. The π-pulse length t_π is fixed at 24 ns with the interval between every adjacent pulse fixed at $\tau_0 = 18$ ns. The coherence decay is measured via increasing the number of decoupling pulses N. Source data are provided as a Source Data file.

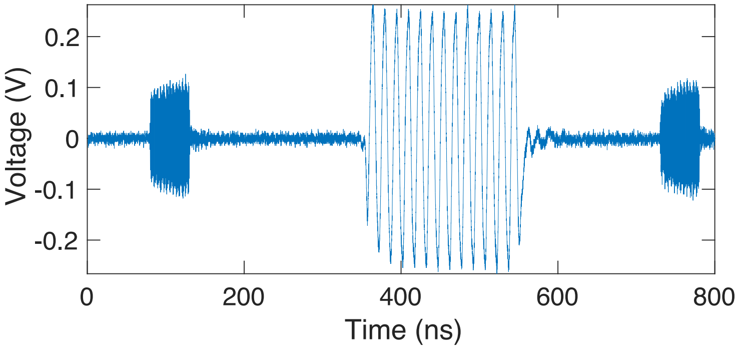

Supplementary Fig. 8. **Characterization of Microwave and RF power** Measured microwave and RF waveform after the stripline measured using an oscilloscope. Here we intentionally extend the microwave and RF pulse length to better quantify the amplitude ratios between the two drives. A 30 dB broadband RF and microwave attenuator is connected to the input of the oscilloscope for protection. Source data are provided as a Source Data file.

(a)

	A_{xx}	A_{yy}	A_{zz}	A_{xy}
N^1_{DFT}	-65.856	-126.294	-65.900	0.000
N^2_{DFT}	-111.396	-81.605	-65.900	25.800
N^3_{DFT}	-111.396	-81.605	-65.900	-25.758
N^1_{adj}	-13.720	-26.322	-65.900	0.000
N^2_{adj}	-23.208	-17.001	-65.900	5.375
N^3_{adj}	-23.208	-17.001	-65.900	-5.366

(b)

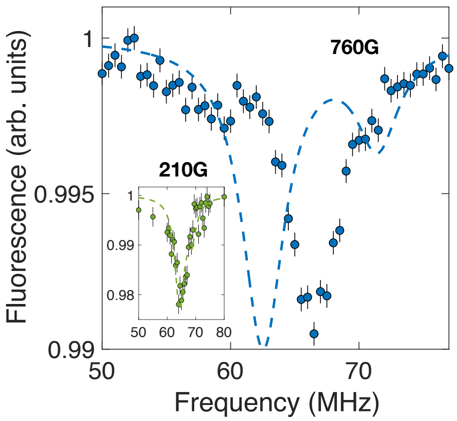

Supplementary Fig. 9. **Nuclear Spin Resonance Measurement** (a) Hyperfine parameters **A** of the three nearest ^{15}N nuclear spins. The first three rows are from ab-initio calculations [4, 5]; the last three rows list the adjusted values by rescaling the transverse components (A_{xx}, A_{yy}, A_{xy}) by a factor of 4.8. (b) ^{15}N nuclear spin resonance spectra at 760 G and 210 G (Inset). Dashed lines show the numerical simulations using the ab-initio predicted hyperfine values. Error bars represent 1 s.d. accounting statistical uncertainties. Source data are provided as a Source Data file.

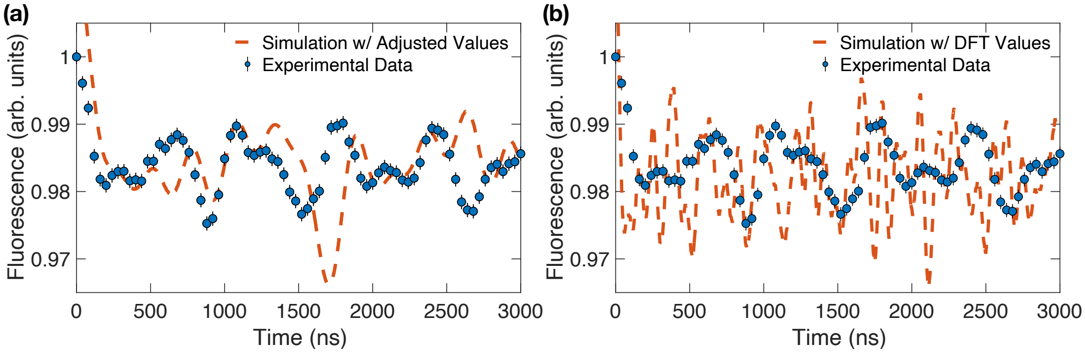

Supplementary Fig. 10. **Nuclear Spin Rabi Oscillations** Experimental data (blue points) superimposed with (a) a nuclear Rabi simulation using rescaled transverse hyperfine coupling strength and (b) the transverse hyperfine strength predicted by ab-initio calculations. The simulation using rescaled hyperfine parameters shows far better agreement with experiment but does not exactly match the trajectory due to unknown degrees of freedom in the angle θ (set to be 0 for simplicity) and the specific values of A_1^j and A_2^j. Error bars represent 1 s.d. accounting statistical uncertainties. Source data are provided as a Source Data file.

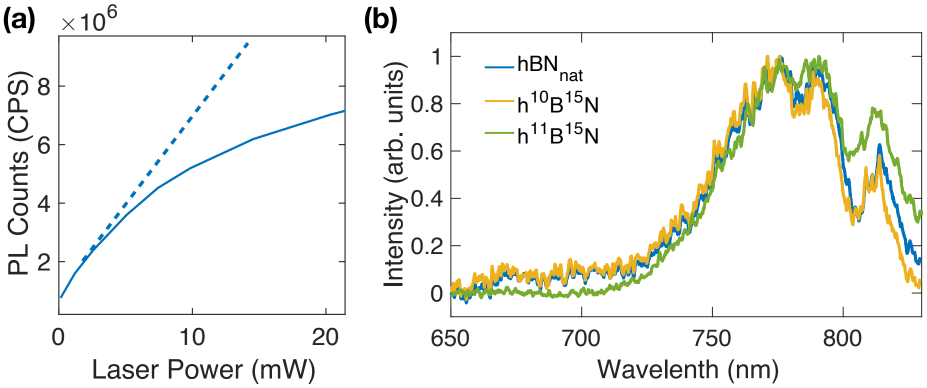

Supplementary Fig. 11. **Optical Characterization** (a) Optical saturation curve of V_B^- in $h^{10}B^{15}N$ against laser power. (b) Photoluminescence spectrum of V_B^- in hBN_{nat}, $h^{10}B^{15}N$, and $h^{11}B^{15}N$. Source data are provided as a Source Data file.

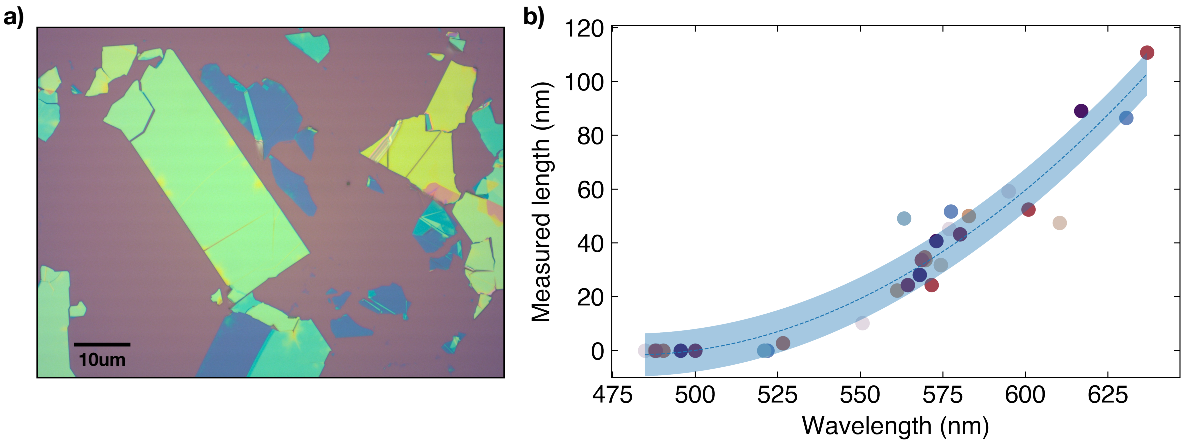

Supplementary Fig. 12. **Thickness calibration curve** (a) A common optical microscopy image of varying thickness hBN flakes on Si wafer with 300 nm thermal oxide. (b) Plotting of AFM thickness and wavelength estimation from optical image. The dashed line shows the second degree polynomial fitting. The shadowed region indicates the standard derivation of $\sigma \sim$8nm. The second degree polynomial fitting shown in the figure was then used for estimating the sample thickness. Source data are provided as a Source Data file.

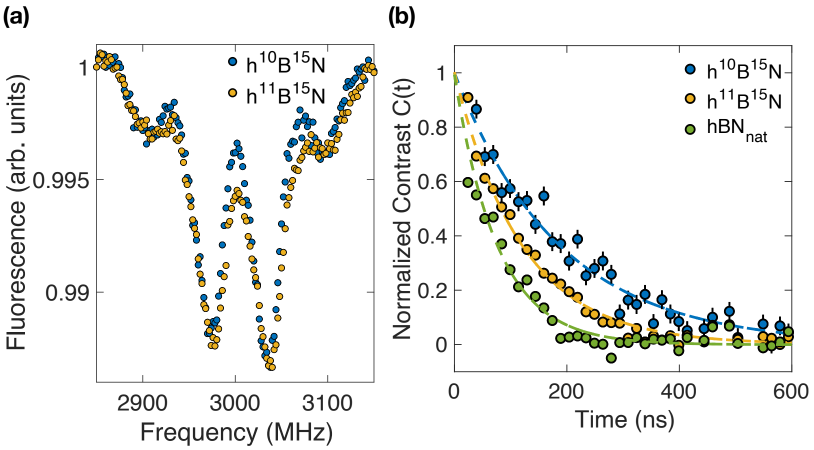

Supplementary Fig. 13. $h^{11}B^{15}N$ **characterization**. (a) The ESR spectra of V_B^- in $h^{10}B^{15}N$ and $h^{11}B^{15}N$. (b) The spin echo measurement. $T_2^E \approx 186 \pm 22,\ 119 \pm 6,\ 76 \pm 7$ ns for $h^{10}B^{15}N$, $h^{11}B^{15}N$, and hBN_{nat} respectively. Error bars represent 1 s.d. accounting statistical uncertainties. Source data are provided as a Source Data file.

[1] R. Gong, G. He, X. Gao, P. Ju, Z. Liu, B. Ye, E. A. Henriksen, T. Li, and C. Zu, Nature Communications **14**, 3299 (2023).
[2] A. Dréau, M. Lesik, L. Rondin, P. Spinicelli, O. Arcizet, J.-F. Roch, and V. Jacques, Physical Review B **84**, 195204 (2011).
[3] J. F. Barry, J. M. Schloss, E. Bauch, M. J. Turner, C. A. Hart, L. M. Pham, and R. L. Walsworth, Reviews of Modern Physics **92**, 015004 (2020).
[4] V. Ivády, G. Barcza, G. Thiering, S. Li, H. Hamdi, J.-P. Chou, Ö. Legeza, and A. Gali, npj Computational Materials **6**, 41 (2020).
[5] X. Gao, S. Vaidya, K. Li, P. Ju, B. Jiang, Z. Xu, A. E. L. Allcca, K. Shen, T. Taniguchi, K. Watanabe, *et al.*, Nature Materials **21**, 1024 (2022).
[6] S. Puebla, H. Li, H. Zhang, and A. Castellanos-Gomez, Advanced Photonics Research **3**, 2100221 (2022).
